# A Framework of Human-Motion Based Structural Dynamics Simulation Using Mobile Devices

**DOI:** 10.3390/s19153258

**Published:** 2019-07-24

**Authors:** Hyungchul Yoon, Kevin Han, Youngjib Ham

**Affiliations:** 1 School of Civil Engineering, Chungbuk National University, Cheongju 28644, Korea; 2 Department of Civil, Construction, and Environmental Engineering, North Carolina State University, Raleigh, NC 27695, USA; 3 Department of Construction Science, Texas A&M University, College Staion, TX 77843, USA

**Keywords:** structural dynamics, educational tool, mobile devices, motion-based

## Abstract

Due to the nature of real-world problems in civil engineering, students have had limited hands-on experiences in structural dynamics classes. To address this challenge, this paper aims to bring real-world problems in structural dynamics into classrooms through a new interactive learning tool that promotes physical interaction among students and enhances their engagement in classrooms. The main contribution is to develop and test a new interactive computing system that simulates structural dynamics by integrating a dynamic model of a structure with multimodal sensory data obtained from mobile devices. This framework involves integrating multiple physical components, estimating students’ motions, applying these motions as inputs to a structural model for structural dynamics, and providing students with an interactive response to observe how a given structure behaves. The mobile devices will capture dynamic movements of the students in real-time and take them as inputs to the dynamic model of the structure, which will virtually simulate structural dynamics affected by moving players. Each component of synchronizing the dynamic analysis with motion sensing is tested through case studies. The experimental results promise the potential to enable complex theoretical knowledge in structural dynamics to be more approachable, leading to more in-depth learning and memorable educational experiences in classrooms.

## 1. Introduction

Due to the nature of real-world problems in civil engineering, students have had limited hands-on experiences in classrooms (e.g., learning concepts of structural behaviors of a bridge on a piece of paper if not on a computer screen). In an attempt to address such a challenge, several studies including [1] have studied the benefits of game-based learning, which better supports modeling and simulation of real-world problems in classrooms than traditional educational media does. Such games include many characteristics of problem-solving (e.g., having multiple paths to a given goal and collaboration among multiple players). Scenarios laying out problems are placed within a gaming framework. For instance, a series of pre- and post-experimental tests taken by diverse control and experimental groups over multiple years reported that applying educational games to lectures on structural concrete design could contribute to improved student performance [2,3,4].

Traditionally, there have been physical games to improve student understanding, e.g., a spaghetti bridge competition where students design and build a truss bridge using spaghetti rods and adhesives. Over the past few years, there have been advances in game-based education tools for structural engineering available online or in the form of smartphone applications. For example, online games, such as “Cargo Bridge” [5] and “Bridge Builder” [6], ask users to build trusses that can withstand predefined loads, given a budget and resources. These games highlight structural members that take critical loads while different loads are virtually applied. Users are able to check where the forces are being distributed over the structures they create. With recent advances in mobile computing and embedded sensors, students have full access to affordable mobile devices that can be utilized for interactive in-class exercises. There have been several smartphone applications for game-based education that teach structural engineering concepts of reaction, shear, moment, and deflection. In such smartphone applications, users are asked to enter lengths and loadings for the structural design of bridges (e.g., simply supported beam), and then the smartphone applications compute reactions, shear and moment diagrams, and deflections of the structures under the given scenarios (e.g., “Bridge Construction Simulator 3D” [7], “Bridge Builder Simulator” [8], or “Civil Engineering Calculations” [9] available in the apple app store).

Despite the benefits of such educational tools available online or in the form of smartphone applications, they limit students’ involvement to design simple structures by feeding a few input values, which prevents them from providing engaged learning experiences for students through bi-directional interaction. Providing an interactive learning environment through physical interaction of students in classrooms can significantly motivate students and further increase their performance [10]. The 2025 Vision from American Society of Civil Engineers has projected that civil engineers will rely more on real-time access to living databases, sensors, diagnostic tools, and other advanced technologies for making informed decisions by 2025 [11]. However, there is still a lack of effort to increase students’ dynamic engagement and interaction by implementing these advanced technologies in the traditional courses that cover fundamental concepts of structural engineering. Moreover, the authors at their current and past institutions have always observed large numbers of unmotivated students who have had very limited chances to learn about advanced technologies that can potentially increase their interests and, therefore, motive them. There have been efforts to increase engagement and interaction in engineering classes through computer-based learning environments via virtual reality (VR) and augmented reality (AR) [12,13,14,15]. Despite their benefits, these prior works have been mostly limited to better visualization of course materials in a passive manner, rather than having interactive components that dynamically involve students in classrooms. Prior works including Holzinger and Ebner [16] and Kettanurak et al. [17] reported that students consider the interactivity (i.e., bi-directional engagement) as the most important element of effective learning. In this sense, there is a need for more interactive learning tools that enable students to immerse themselves in a realistically simulated class setting for improved engineering education.

The interactive learning environments, that the proposed system can provide, will enable complex theoretical knowledge to be more approachable, leading to more in-depth learning and memorable educational experiences. Traditional in-class example problems or demonstrations are very limited to passive activities, especially in structural dynamics where structural motions are time-dependent. In an effort to address these challenges, this paper focuses on creating and validating a new educational gaming framework that connects multiple mobile devices, captures students’ dynamic movements, solves the equation of motion of the dynamic system, and visualizes the resulting structural behaviors in real-time to provide an interactive learning experience to students. The proposed game-based approach can bring large-scale structures into classrooms and animate their response in real-time rather than static illustrations. The proposed work is driven by the hypotheses that students will better understand engineering concepts through physical interactions that provide engaged learning [10,18,19]. This paper mainly focuses on the development and testing of the proposed system that integrates cyber physical components (i.e., computational algorithms, mobile devices, and a computer). The computational algorithms include a dynamic model, wireless network configuration, and dead-reckoning, and the physical components involve mobile devices and a processing computer (i.e., an instructor’s laptop).

The main contribution of this paper to the body of knowledge in computing in civil engineering is to develop and test a new interactive computing system that simulates structural dynamics by integrating a structural model with multimodal sensory data obtained from mobile devices. This system includes (1) integration of multiple physical components, (2) analysis of their inputs, (3) applying them as inputs to a structural model for structural dynamics, and (4) providing users with an interactive response to observe how a given structure behaves. The mobile devices will capture dynamic movements of the users in real-time and take them as inputs to the structural model, which will virtually simulate structural dynamics affected by moving players. This tool will help students better understand how structures behave in a dynamic loading condition (i.e., moving users), illustrating structural dynamics. The students will, in near real-time, see how a structure behaves as they walk and jump around a classroom.

## 2. System Development

### 2.1. Overview

**Figure 1:**
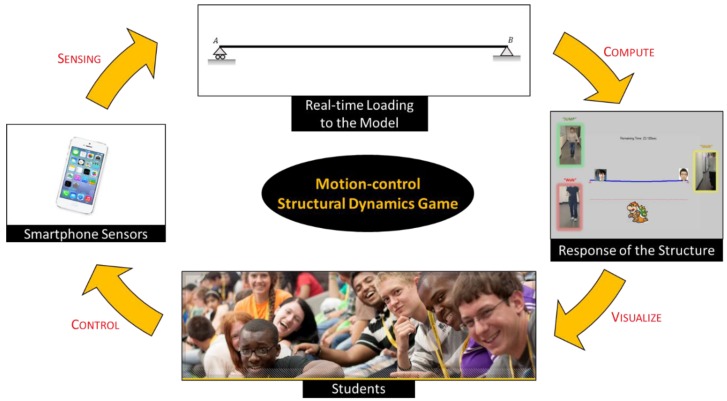
The proposed interactive learning tool for structural dynamics.

We transfer sensing data obtained from mobile devices to a server in real-time and then leverage a structure model for structural dynamics. Android smartphones are used for sensing purposes, and the Transmission Control Protocol/Internet Protocol (TCP/IP) connection is chosen as the communication mechanism between embedded sensors and the server. We use three sensors embedded in mobile devices: accelerometer, gyroscope, and magnetic field sensor (i.e., compass). We first create a dynamic model of a structure in Simulink with a predefined dynamic live load. Then, the predefined loading is continuously updated with dynamic loads as game players virtually affect the dynamics of the structure (Figure 1).

**Figure 2:**
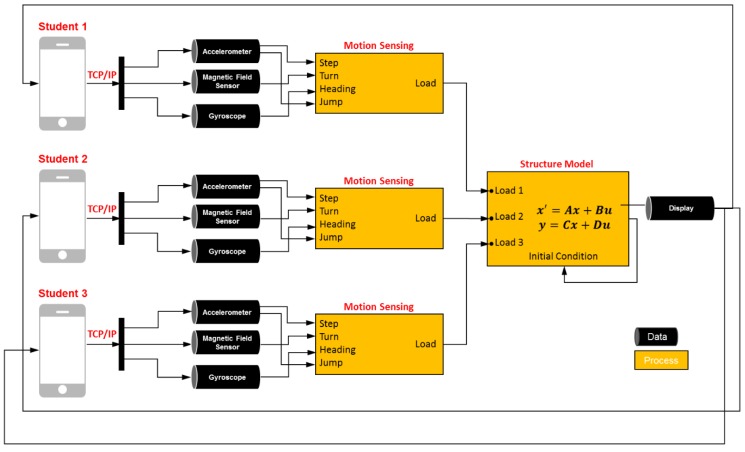
Overview of the proposed system.

Figure 2 illustrates an overview of the proposed computing system. The overall system consists of (1) collecting sensory data from accelerometers, gyroscopes, and compasses embedded in mobile devices; (2) recognizing steps and jumps made by each player, and inferring the directions of the players’ movement; (3) applying live loads to the structural model based on players’ movements, and simulating the resulting structural responses in the structure model; (4) visualizing the dynamic movement of the structure; and (5) enabling the game players to immerse themselves in a realistic simulated class setting. This process is repeated over time and can be performed with multiple players in real-time. In the following sections, the underlying algorithms building on the embedded sensors in mobile devices are presented. Then, the experimental results on several case studies, a usage scenario of how the developed tool can be implemented in a structural engineering classroom, perceived benefits, and open research challenges are discussed in detail.

### 2.2. Data Collection

**Figure 3:**
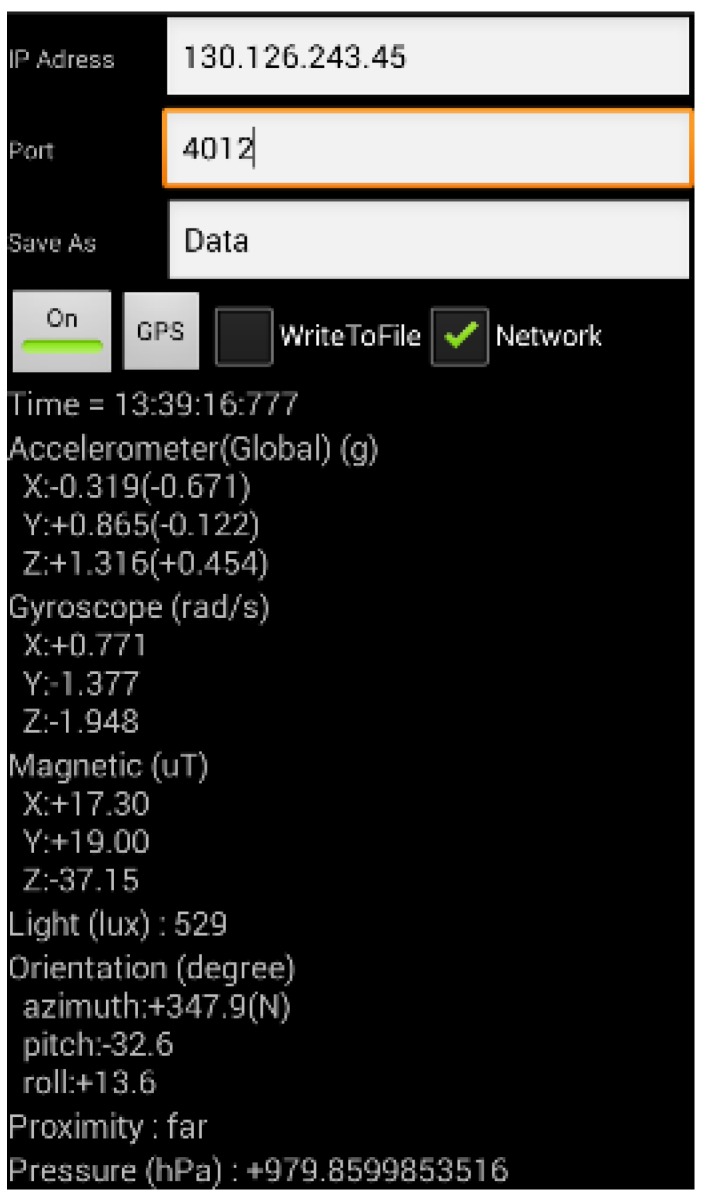
Android application for data collection.

In this paper, we developed an Android application (denoted throughout as an app) that collects sensory data (see Figure 3). The developed app collects (1) acceleration, (2) magnetic field, and computes (3) the orientation of the phone using the gravity vector with the magnetic field over time. The app transmits the obtained sensory data to a PC server using the TCP/IP to simulate resulting structural dynamics in the following step. The app enables encoding of the data to a comma-separated values (CSV) file and direct transferring of the data to the server.

### 2.3. Motion Sensing

**Figure 4:**
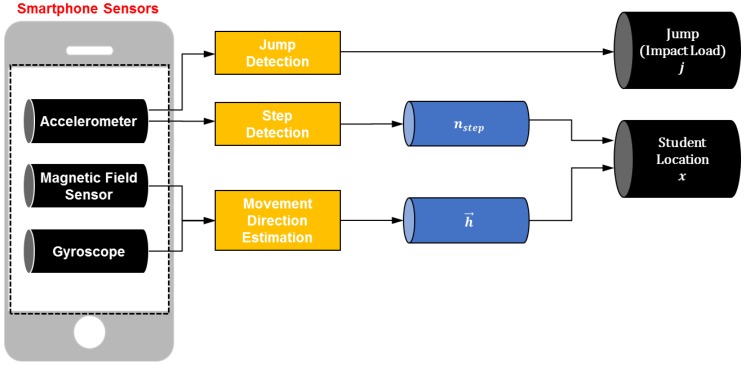
Overview of motion sensing using smartphones.

Localizing game players and recognizing their activities in real-time are two of the most critical parts in the proposed interactive learning tool. Generally, GPS is the most widely used method for the localization of mobile devices. However, the GPS module is inefficient in terms of energy consumption and performs poorly in indoor environments. Building upon the motion sensing system by Yoon et al. [20], we developed a dead-reckoning method to estimate one’s location to address challenges in localization technologies such as GPS, UWB, and WLAN that are most likely reliable in outdoor environments. This method finds one’s current position relative to the previous location and advances the position over a given time period, which is infrastructure-free and yields outcomes at a reasonably practical range of accuracy as recently validated in [20]. This phase is composed of the following two modules: (1) step and jump detection and (2) movement direction estimation (see Figure 4). Using both modules, the location of game players and where they jump can be estimated, which will be later used as the input live loading to the structural dynamics. The following sections describe each module in detail.

#### 2.3.1. Step & Jump Detection

**Figure 5:**
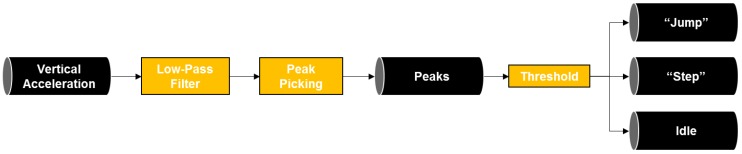
Step and jump detection.

The step and jump detection module leverages sensory data from accelerometers in a mobile device to detect the moments of steps and jumps made by the game players. The procedure of the step and jump detection is shown in Figure 5. First, a low-pass filter is applied upon vertical acceleration to eliminate noise in the sensory data. To remove false positive detection of steps, negative and positive peaks are chosen from the data, and only the peaks that have a higher value of negative and positive peak difference were selected as a step. Here, the threshold value is empirically selected to capture the step and jump activities. For the reported experiments in this paper, the threshold for the step and jump detection was set to 0.2 of the maximum peak based on the empirical founding. A jumping activity typically creates residual vibrations that may result in false positive detections (i.e., detecting residuals as steps or jumps). To minimize such misclassifications, the jump detection algorithm is forced to pause for 0.5 s (based on the empirical founding in the case studies) whenever it detects the jump activity.

#### 2.3.2. Movement Direction Estimation

**Figure 6:**
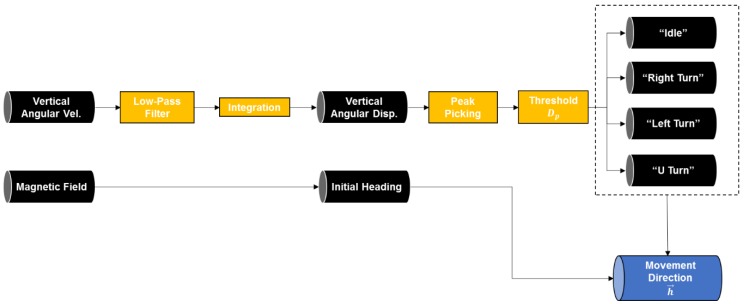
Overview of estimating the players’ movement directions.

The module that estimates directions of user’s movement uses a gyroscope and a magnetic field sensor of a mobile device to detect the player’s moving direction. The procedure of the turn detection is shown in Figure 6. First, a low-pass filter is used to eliminate the noise in the data. Second, the numerical integration is applied to obtain the angular displacement. However, due to the drift errors of the gyroscope displacement, the angular displacement alone is most likely unreliable to determine one’s direction of movement. To achieve better accuracy in the direction estimation, both positive and negative peaks are selected, and the negative peak is compared with the next positive peak to determine if a turn is made. Finally, the differences between these two peaks (Dp) are used to determine “Left Turn”, “Right Turn”, “U-Turn”, and “Go Straight (Idle)” as shown in Table 1. Once the turn is identified, the directions of the game players’ movement can be determined by combining the detected turn with the initial heading information obtained by the magnetic field sensor as

(1) $${\vec{h}}_{t}={\vec{h}}_{0}+\overset{t}{\underset{i=1}{\sum }}T_{i}$$

where ${\vec{h}}_{t}$ is the current heading at time t, ${\vec{h}}_{0}$ is the initial heading direction obtained by the magnetic field sensor, and $T_{i}$ is the detected turn information obtained by Table 1.

### 2.4. Structure Dynamics Analysis Based on Motion Sensing

**Figure 7:**
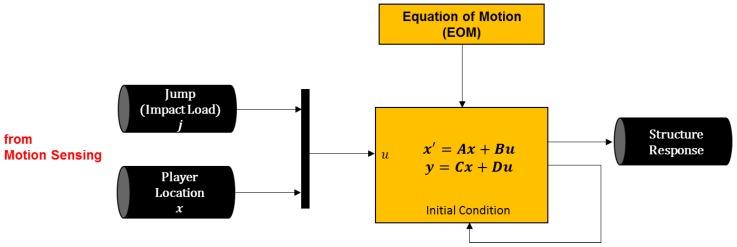
Overview of the structural dynamic analysis.

Once the locations of game players and where each player made jumps are identified through motion sensing, this information is converted into the live load condition to the dynamic structure model (Figure 7). Here, the location of each game player is acting as a moving load, and the jump activities of the players are considered as an impact load to the given structure at the location of each player. Here, various types of structural models can be automatically generated depending on the user-specified parameters. For example, a user can change the boundary condition of the beam; properties of the material and geometry; as well as the calculation parameters, such as the number of sections.

#### 2.4.1. Equation of Motion (EOM)

The equation of motion for a structural system using assumed modes can be written as

(2) $$M\ddot{q}\left(t\right)+C\dot{q}\left(t\right)+\mathrm{Kq}\left(t\right)=\mathrm{GF}$$

where M is the mass matrix, C is the damping matrix, K is the stiffness matrix, and G is the force matrix.

Assuming n modes, the shape functions $\psi_{1}\left(x\right)$, $\psi_{2}\left(x\right)\ldots \psi_{n}\left(x\right)$ are selected by imposing appropriate boundary conditions to the model (e.g., for simply supported beam, $\psi_{1}\left(x\right)=\sin\left(\frac{\pi x}{L}\right),\;\psi_{2}\left(x\right)=\sin\left(\frac{2\pi x}{L}\right),\;\ldots ,\;\psi_{n}\left(x\right)=\sin\left(\frac{2nx}{L}\right)$). The mode shape matrix ψ is then constructed by combining all mode shapes. The stiffness matrix  K and the mass matrix M are calculated based on the assumed mode method as below, and the damping ratio ζ is assumed to be 2%.  E is the elasticity of the modulus of the beam, I is the moment of inertia, ρ is the density, and A indicates the section area of the given structure.

(3) $$K=\int_{0}^{L}EI\Psi^{\prime \prime }{}^{T}\;\Psi^{\prime \prime }\;dx$$

(4) $$M=\int_{0}^{L}\rho A\Psi^{T}\Psi dx$$

Using the two matrices, the natural frequency $\omega_{n}$ and the mode shape  Φ can be determined using the eigenvalue. The modal matrices $M_{r}$, $K_{r}$ and $C_{r}$ can be calculated using the mode shapes. Then, the damping matrix C can be calculated by using the equations below.

(5) $$\;[\omega_{n}^{2},\;\Phi ]=\mathrm{eig}\left(K,M\right)$$

(6) $$M_{r}=\Psi^{T}M\Psi$$

(7) $$K_{r}=\Psi^{T}M\Psi$$

(8) $$C_{r}\left(i,i\right)=2\zeta \sqrt{M_{r}\left(i,i\right)K_{r}\left(i,i\right)}$$

(9) $$C=\Psi^{-T}C_{r}\Psi^{-1}$$

#### 2.4.2. State Space Representation

The constructed model can be represented as the state space to be used in Simulink. By selecting the displacement, velocity, and accelerometer as the output of the system, each of the system matrices can be represented as follows:

(10) $$\dot{x}=\;[\begin{array}{c} \dot{q}\\ \ddot{q} \end{array}]=A_{s}\;[\begin{array}{c} q\\ \dot{q} \end{array}]+B_{s}F$$

(11) $$y=\;[\begin{array}{c} \begin{array}{c} q\\ \dot{q} \end{array}\\ \ddot{q} \end{array}]=C_{s}\;[\begin{array}{c} q\\ \dot{q} \end{array}]+D_{s}F$$

(12) $$A_{s}=\;[\begin{array}{cc} 0 & I\\ -M^{-1}K & -M^{-1}C \end{array}],{\;B}_{s}=\;[\begin{array}{c} 0\\ -G \end{array}],\;C_{S}=\;[\begin{array}{cc} I & 0\\ 0 & I\\ -M^{-1}K & -M^{-1}C \end{array}],\;D_{s}=\;[\begin{array}{c} 0\\ 0\\ -G \end{array}]$$

#### 2.4.3. Combining with Motion Sensing

**Figure 8:**
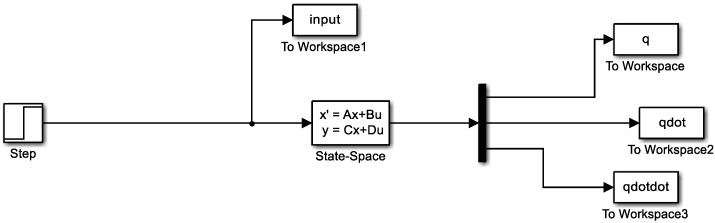
Simulink model for the structure system.

Figure 8 illustrates the Simulink model of the structural system. All the matrices listed here except the force matrix **G** are time-invariant matrices because the structure itself is assumed not to change over time. However, the input load changes over time (e.g., location and amplitude) and, therefore, is a time-variant matrix. The output of the system for each time step is assigned as the initial condition of the next time step, and the system calculates the structural response using the updated input matrix **G**. The force matrix **G** is updated every time to reflect the location and amplitude of the dynamic loads, as shown in Equation (13). The magnitude of the load, α, is determined by whether the game players step, jump, or are idle. $x_{L}$ is the location of the load determined by the dead-reckoning-based localization module.

(13) $$G=\alpha *\rho A\Psi^{T}\left(x=x_{L}\right)$$

The output of the state-space model y will be a vector of displacement, velocity, and acceleration of q. The displacement of the given structure v at location x can be obtained by multiplying the shape function ψ(x)  as below.

(14) v=q*ψ(x)

## 3. Experiments

### 3.1. Experimental Setup and Results

**Figure 9:**
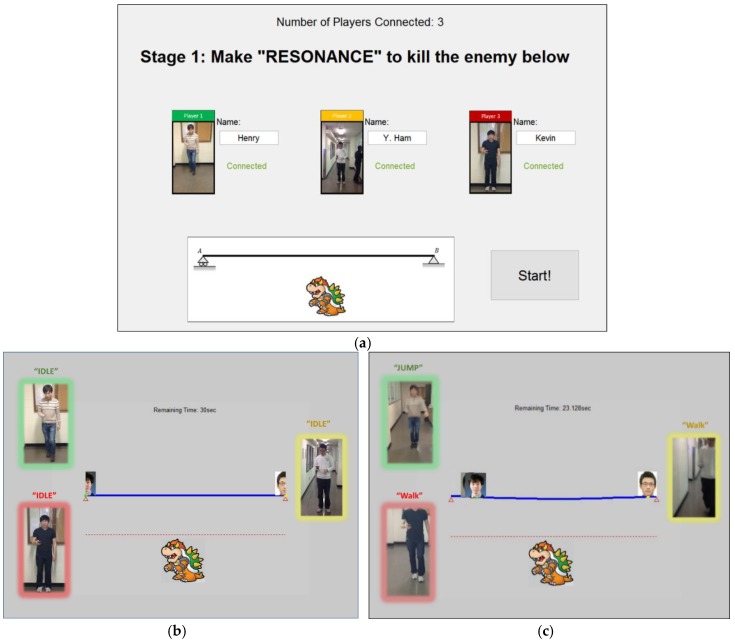
(**a**) Initial screen of the system for setup; (**b**) play screen at time = 0; (**c**) play screen with three moving users.

**Figure 10:**
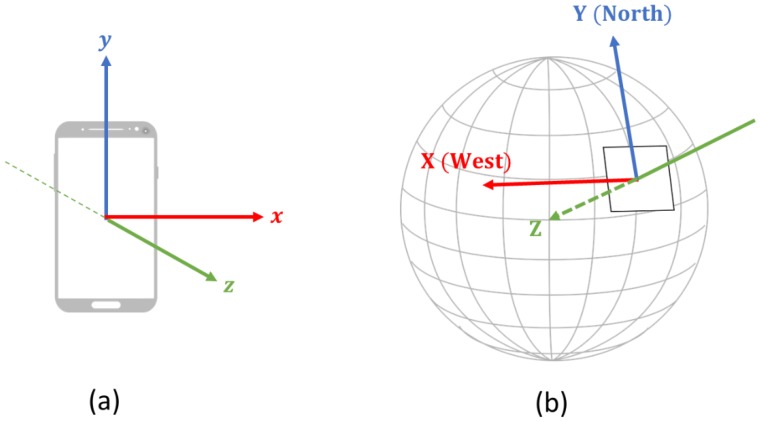
Coordinate systems in (**a**) local device and (**b**) global environment.

**Figure 11:**
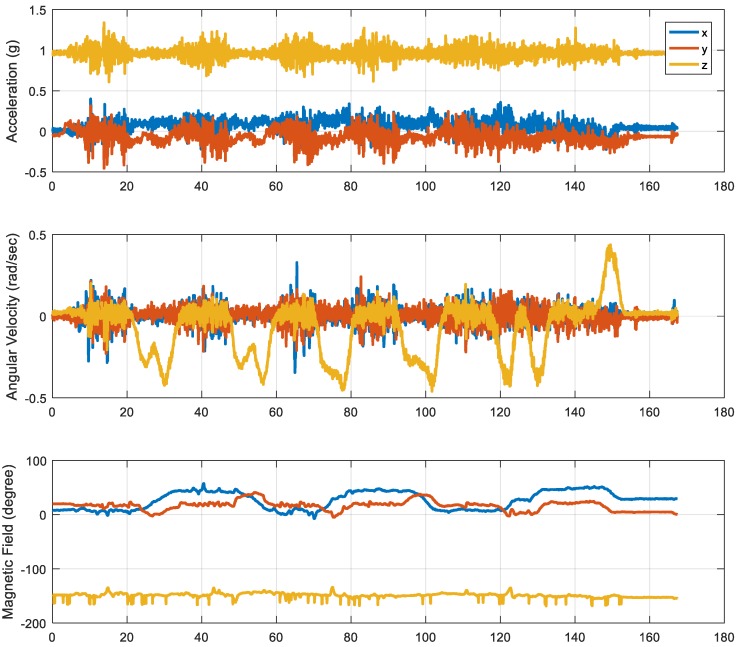
Examples of collected raw sensory data: acceleration (**Top**), angular velocity (**Middle**), and magnetic field (**Bottom**).

Figure 9 shows the screenshot of the initial and play screens. In these experiments, each of the three participants holding a smartphone (Samsung Galaxy S4) in their hands were connected to the system, and the game began when a start button was pressed. Once the game started, acceleration, angular velocity, and magnetic field sensor data were collected at the sampling rate of 10 Hz from each smartphone by using the data collection app (Figure 3). The sensory data obtained from smartphones is in the local device coordinate system of the smartphone as shown in Figure 10a. The local device coordinate system is defined as the x-axis to be horizontal, y-axis to be vertical, and z-axis to be perpendicular to the phone. Since each user may use smartphones at a different orientation, the sensory data was transformed into the global coordinate system using the approach similar to Yoon et al. [21]. The global coordinate system is defined as the X-axis to be west, Y-axis to be north, and Z-axis to be toward the center of the Earth. Finally, the data were transmitted to the server (Figure 11).

**Figure 12:**
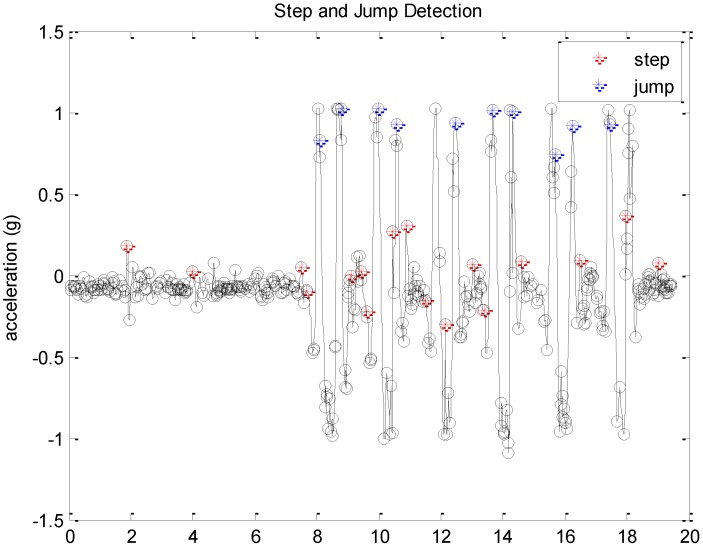
An example of step and jump detection from accelerometer data.

To evaluate the proposed interactive learning tool for structural dynamics, a series of experiments were conducted, recognizing the game player activities in classrooms (i.e., walking, turning, and jumping) based on sensory data obtained from the device. We examined the accuracy of step, turn, and jump detection with three different players. This accuracy gives an indication of how precisely the game players’ actions can be localized in the given structure model, which relates to the error between the actual locations of the game players equipped with smartphones and the location virtually mapped to the structure model as dynamic live loads. In our experiments, a total of 121 steps and 18 jumps were detected, and the averaged accuracy of step and jump detection with three different players were 96.7% and 100% respectively. Figure 12 shows an example of the step and jump detection results by using the acceleration data. Whenever the users make steps and/or jumps, the acceleration data showed one or more peaks corresponding to the step and/or jump activities. By applying the proposed activity detection approach, we were able to eliminate most of the false-positive detection.

**Figure 13:**
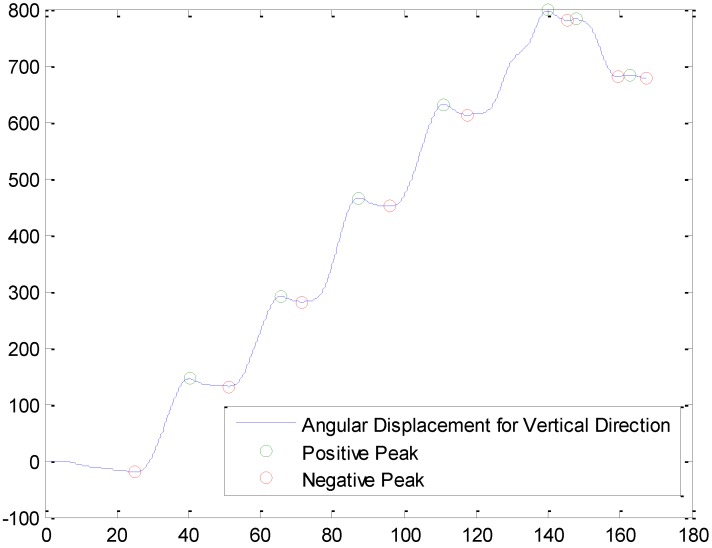
An example of turn detection from a gyroscope and a magnetic field sensory data.

Figure 13 shows an example of turn detection in the case studies. Here, the game player has made five left turns and one right turn. The positive peaks and negative peaks are marked as circles upon the angular displacement. For example, when the player made a U-Turn, the difference in the value between the first red circle and the next green circle was 160° which results in “U-Turn” (see Table 1). In our experiments, a total of 21 turns was made, and all turns made by three different game players were successfully captured by calculating the difference between the positive and negative peaks.

### 3.2. Time & Data Synchronization

**Figure 14:**
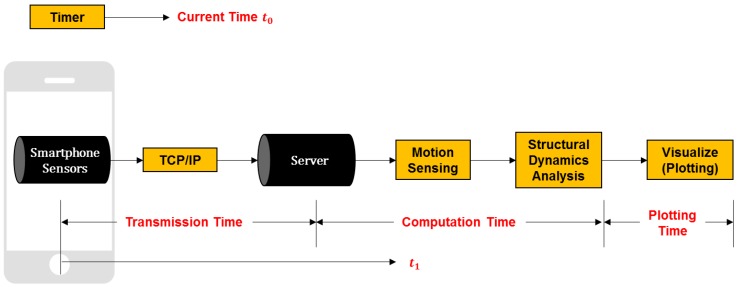
Overview of time and data synchronization.

This performance metric evaluates how accurately the time of the (1) smartphone, (2) Simulink, and (3) structure model are synchronized between each other. To do that, we measured the time required for implementing the following processes: (1) transmitting the sensory data obtained from single or multiple smartphones to the given structure model through the TCP-IP protocol, (2) simulating the corresponding structural behaviors in Simulink, and (3) visualizing the results in the given structure model (Figure 14). In addition to the time synchronization, we evaluated how precisely different data obtained from multiple smartphones can be synchronized between each other. Since we intend to develop the proposed computing tool as a soft real-time system, tasks are not forced to be aborted even though they cannot be completed in time. Rather, the tasks in our system will be completed even when they require more than the available time.

The sampling rate of the sensor was selected as 10 Hz. According to our preliminary data, the dead-reckoning and the structural dynamic analysis require at least three data points, which corresponds to 300 ms of cumulated data. However, for some data segment, the total computing time including transmission, computation, and visualization took more than 300 ms (Table 2). Eventually, the results were slightly lagged as time goes by. To resolve this issue, the adaptive analysis period (Δt) was introduced, so that the lag does not occur. Since the analysis time varies depending on network environments, we measured the current time (t_0_) and the analysis time (t_1_). Then, if the calculation time is behind the current time more than a chosen threshold, it cumulates the data for the next time step without performing the calculation. Here, this cumulated data is batch-processed and does not impact the structural analysis.

## 4. Discussion

The suggested system has been developed as a class support tool for structural dynamics, especially associated with the class objectives such as “what is a natural frequency of a structure?”, “when does resonance occur?”, “what is a mode shape, and why is it important?”, and “what is a damping and how does it affect the response of a structure?”. To help better understand the usage of the developed computing system in the form of an app, the following scenario describes how the proposed learning tool could potentially promote engaged and interactive learning in a structural engineering classroom: It is the second week of the semester in structural dynamics class, and the topic of “natural frequency” will be covered in the class. At the beginning of the class, the instructor asks for three volunteers who will be demonstrating the developed tool as players. Each student launches the application on his or her smartphone, having the device connected to the server (e.g., instructor’s laptop) in the classroom. All students will be able to watch a projector screen with the players virtually standing on a bridge. Now, the instructor asks players to move around and jump to see how the bridge will respond to their impact loading. Whenever the players move, the locations of the loads will be continuously updated, and the impact load will be applied when they jump. A few minutes later, they will be asked to generate the largest dynamic displacement. The players will be jumping in various locations with different frequencies, illustrating how the bridge responds to game players’ motions. Finally, by trial and error, players will find out that the dynamic displacement will be at its maximum when they all jump together, repeating every second. After the demonstration, the instructor resumes the lecture, introducing the concept of the natural frequency. At the end of the class, students will receive the 2-week gaming assignment designed to help them apply their understanding of “Natural Frequency and Mode Shapes.” A team runs the game on their personal computer and smartphones. Once their smartphones are connected to the game by the local network, a beam and an associated dynamic response will appear on their screen. Once the game has started, students will keep changing their locations of the loads to find the loading conditions that satisfy the mission objective. Once their dynamic response is matched with the objective, the stage will be cleared and will move to the next stage. They will continue playing the game until they clear all stages for the given topic. Upon completion of the game, the team will discuss their findings (e.g., how the specific loading conditions will affect the dynamic response of the structure). Finally, the team will be asked to email the result of the game together with the report to the instructor. The team members now have a better understanding of how a structure will respond to a specific dynamic load by the completion of this gaming assignment.

## 5. Conclusions

This paper presents an interactive learning tool for teaching structural dynamics concepts. The developed tool provides physical interaction among students in classrooms. The tool is especially useful to help students with limited background in math and engineering understand the concepts of structural dynamics. The underlying system is building on a dynamic model that reacts to moving game players as live loads. The components of synchronizing the dynamic analysis with motion sensing are tested through case studies. Since this paper mainly focuses on the algorithmic development of the proposed interactive learning tool and the technical validation, evaluating the student performance is reserved for future works. The results also show the potential development of other applications using the proposed concept. The following are what we envision as possible applications: (1) We envision that the foundation of this research can be used for large numbers of other applications that require live load estimation in real-time. For instance, one possible application is to simulate how a railroad bridge that a train passed by should behave. This information can be compared to what sensors tell about how it behaves, providing valuable information about the current structural states of the bridge. Another application would be the safety analysis of construction workers working on top of temporary structures by simulating its stress level and the displacement. This would be useful as it is unlikely to have a structural health monitoring system installed for temporary structures in jobsites: and (2) the developed tool can easily turn into games to be used for outreach activities. For instance, K-12 students can play a game that breaks a bridge by jumping around and creating loading conditions that the bridge cannot withstand. To provide entertainment, we can design the game to place an enemy below the bridge (e.g., Bowser in Figure 9) and have them create the largest deflection at the location of where the enemy is standing and crush the enemy. These games can be used during open-house events at educational facilities or visits to K-12 schools to increase student interest in structural engineering. While this paper shows the potential of using mobile devices to assist in the learning of structural dynamics, future works involve: (1) the current system is based on a threshold-based model with filters, and the threshold value is empirically selected. Thus, the threshold values are changed depending on the location (whether the phone is in a pocket or held by hand) and the type of the mobile device. Providing a device-invariant or location-invariant threshold value will enhance the flexibility of this framework; and (2) the proposed system builds on a simple beam model with assumed mode, but the current dynamic model would be replaced with any FE model, such as an existing bridge or building, which would be more attractive for the learning of structural dynamics. All these are currently being explored as part of our ongoing research.

## Figures and Tables

**Table 1:** Turn types based on the ranges of Dp.

Range of D_p_	Turn (T)
−135 < D_p_ < −45	Right Turn
−45 < D_p_ < 45	Idle
45 < D_p_ < 135	Left Turn
135 < D_p_ or −135 > D_p_	U Turn

**Table 2:** Computing time for transmission, computation, and visualization.

Case	Transmission Time (ms)	Computation Time (ms)	Plotting Time (ms)	Overall (ms)
1	9	122	269	400
2	10	109	147	266
3	20	121	151	292
4	13	120	155	288
